# HWANet: A Haar Wavelet-based Attention Network for remote sensing object detection

**DOI:** 10.1371/journal.pone.0330759

**Published:** 2025-09-04

**Authors:** Baohua Jin, Fukang Yin, Wenpeng Cai, Hongchan Li, Haodong Zhu, Wei Huang, Qinggang Wu, Hui Chen, Zhongchuan Sun

**Affiliations:** 1 School of Computer Science and Technology, Zhengzhou University of Light Industry, Zhengzhou, China; 2 Engineering Training Centre, Zhengzhou University of Light Industry, Zhengzhou, China; Hunan Normal University, CHINA

## Abstract

Remote sensing object detection (RSOD) is highly challenging due to large variations in object scales. Existing deep learning-based methods still face limitations in addressing this challenge. Specifically, reliance on stride convolutions during downsampling leads to the loss of object information, and insufficient context-aware modeling capability hampers full utilization of object information at different scales. To address these issues, this paper proposes a Haar wavelet-based Attention Network (HWANet). The model includes a Low-frequency Enhanced Downsampling Module (LEM), a Haar Frequency Domain Self-attention Module (HFDSA), and a Spatial Information Interaction Module (SIIM). Specifically, LEM employs the Haar wavelet transform to downsample feature maps and enhances low-frequency components, mitigating the loss of object information at different scales. The HFDSA module integrates Haar wavelet transform and explicit spatial priors, reducing computational complexity while enhancing the capture of image spatial structures. Meanwhile, the SIIM module facilitates interactions among information at different levels, enabling multi-level feature integration. Together, SIIM and HFDSA strengthen the model’s context-aware modeling capability, allowing full utilization of multi-scale information. Experimental results show that HWANet achieves 93.1% mAP50 on the NWPU VHR-10 dataset and 99.1% mAP50 on the SAR-Airport-1.0 dataset, with only 2.75M parameters, outperforming existing methods.

## Introduction

Remote Sensing Object Detection (RSOD) is the process of using object detection methods to identify and locate various ground objects, such as buildings, roads, vegetation, and water bodies, in remote sensing imagery [1–4]. Due to its significant applications in land use planning, forest resource surveys, and agricultural monitoring, RSOD has become a key research topic in the field of remote sensing [5–7].

However, factors such as imaging angles, terrain fluctuations, and object distances in remote sensing images cause significant scale variations in target objects, which in turn increase the complexity of object detection algorithms [8–10]. Traditional object detection methods [11,12] rely on handcrafted features, such as edges, textures, and colors, for object recognition. These methods, however, perform poorly when handling objects of different scales. In recent years, deep learning methods, particularly Convolutional Neural Networks (CNN), have made considerable progress in RSOD. By automatically learning high-level features from images, deep learning models demonstrate greater adaptability and robustness when encountering large variations in object scales [13,14].

Many researchers have applied deep learning methods to tackle the challenge of object scale variations in RSOD. Liang et al. [15] proposed the Spatial-Channel Dual-Frequency Mixer (SCDFMixer) for RSOD, addressing large variations in object scales through a scale-adaptive perception aggregator (SPA). Lin et al. [16] introduced the Scale Selection Network(SSN), which optimizes object detection performance by selecting multi-scale features to handle the object scale variations in RSOD. The methods mentioned above are based on CNN. However, due to the locality inherent in convolution operations, CNN faces limitations when dealing with objects that exhibit significant scale variations, as they struggle to capture global information effectively. This constraint weakens the model’s context-aware modeling capability. Consequently, researchers have introduced the self-attention mechanism to overcome the limitations of CNN in modeling global dependencies and to improve scale variation handling. Gao et al. [17] proposed a few-shot object detection model for remote sensing images that uses Feature Aggregation and the self-attention mechanism to handle scale variations in objects. Zhou et al. [18] proposed a correlation learning detector based on transformer (CLT-Det), which uses the self-attention mechanism to capture spatial correlations and positional information, addressing the challenge of large variations in object scales.

Although these methods leverage self-attention to effectively capture global information, the separate extraction of global and local features by self-attention and CNN may lead to insufficient feature fusion, thereby affecting the recognition of small targets or detailed features.

To overcome the limitations of CNN and self-attention in handling significant variations in object scale in RSOD, researchers have proposed a method that combines both approaches. Li et al. [19] proposed a Pyramid Convolutional Vision Transformer(PCViT), which captures information about objects at different scales through a pyramid structure and enhances feature extraction capability by introducing a Parallel Convolution Module (PCM). Xue et al. [20] proposed a Dual network structure with InterweAved Global-local feature hierarchy based on the TRansformer architecture (DIAG-TR), which enhances object detection in remote sensing images by combining CNN and self-attention, and capturing object information at different scales through the global-local feature hierarchy. Zhan et al. [21] proposed a novel transformer-based multigranularity visual language fusion (MGVLF) module, addressing the scale variation of objects in RSOD by introducing a combined CNN and Transformer approach, which enhances object localization performance through multi-scale visual features and multi-granularity textual embeddings.

Although the aforementioned methods have made some progress in addressing object scale variations in RSOD, they generally rely on strided convolutions for downsampling, which leads to the loss of information for objects at different scales. While methods combining CNN and self-attention partially alleviate the shortcomings of both, existing studies still overlook the crucial role of explicit spatial priors in self-attention for global information extraction. This results in an insufficient understanding of the spatial structure of the image, making it difficult for the model to accurately capture the spatial relationships between objects of different scales, thereby limiting its context-aware modeling capability. Furthermore, these methods lack effective mechanisms for multi-level feature interaction, failing to fully explore the relationships between local and global information, leading to conflicts among feature information and further restricting the effectiveness of context-aware modeling.

To solve the aforementioned issues, this paper presents a Haar wavelet-based Downsampling and Attention Network (HWANet). First, we design a Low-frequency Enhanced Downsampling Module (LEM) to replace traditional strided convolutions. This module employs the Haar wavelet transform [22] to downsample feature maps and enhance low-frequency information, thereby achieving effective downsampling and preventing information loss. Subsequently, to improve the model’s context-aware modeling capability and enable it to fully utilize information from objects at different scales and their related features, we design two modules: Haar Frequency Domain Self-attention(HFDSA) and Spatial Information Interaction Module(SIIM). Specifically, HFDSA incorporates explicit spatial priors when extracting global information, thereby improving the model’s understanding of spatial structures within the image. Meanwhile, SIIM effectively integrates multi-level feature information by strengthening interactions between features of different levels during fusion.

This paper makes the following contributions:

- The Haar wavelet-based Downsampling and Attention Network (HWANet) is proposed to improve the performance of RSOD under large variations in object scales. Compared to other models, HWANet demonstrates superior performance.
- The Low-frequency Enhanced Downsampling Module (LEM) is designed, which utilizes the Haar wavelet transform to downsample feature maps and enhance the decomposed low-frequency components. This effectively prevents the loss of information for objects at different scales during the downsampling process.
- The Haar Frequency Domain Self-attention Module (HFDSA) is designed. By incorporating explicit spatial priors, this Module enhances the model’s understanding of spatial relationships between objects at different scales in the image, thereby improving the model’s context-aware modeling capability. Meanwhile, integrating the Haar wavelet transform significantly reduces the computational complexity of the self-attention.
- The Spatial Information Interaction Module (SIIM) is proposed. By reinforcing the interaction among information at different levels, it effectively integrates multi-level features, and reduces conflicts between features, thereby enhancing the model’s context-aware modeling capability.

## Related works

### Remote sensing object detection model

Currently, deep learning-based RSOD models are mainly divided into two categories: two-stage detection models and one-stage detection models.

The research on two-stage object detection models has been widely explored in remote sensing scenarios. For example, Shi et al. [23] proposed a dual-head global reasoning network (DGRN) that combines classification and localization by propagating visual and spatial embeddings between positive and negative candidate regions, and performing relational reasoning with a graph convolutional network. Liu et al. [24] proposed ORFENet, which integrates object reconstruction and a multi-receptive field adaptive feature enhancement module (MRFAFEM) to reduce object information loss during training and dynamically enhance the micro-object detection capability through multi-receptive field features. Although two-stage detection methods have certain advantages in accuracy and localization, their overall process is relatively complex, computationally intensive, and slower in detection speed. They require generating candidate regions first, followed by feature extraction, region classification, and bounding box regression.

One-stage detection models directly perform class prediction and bounding box regression on the image, eliminating the need for candidate region generation, thus achieving an end-to-end training process and faster detection speed. Based on the one-stage YOLO model, Yi et al. [25] proposed LAR-YOLOv8, an improved RSOD model based on YOLOv8. This model uses a dual-branch attention mechanism to enhance feature extraction and combines attention-guided bidirectional feature pyramid networks and a robust RIOU loss function to improve the model’s detection accuracy. Zhang et al. [26] proposed FFCA-YOLO, a model for small object detection, which enhances feature extraction capability through feature enhancement methods and reduces background interference in remote sensing images. Wang et al. [27] proposed a unified framework called Feature Fusion Single-Stage Detection (FMSSD), which strengthens contextual information by performing feature fusion at multiple scales and within the same-scale layers, thus balancing detection speed and accuracy.

RSOD typically requires strong real-time performance and low computational demands. Therefore, although two-stage detection models have certain advantages in accuracy, their complex multi-stage process and large computational load often make it difficult to meet the requirements of real-time applications. Based on this, this paper uses the one-stage object detection model YOLOv8 as the baseline model for this study.

### Remote sensing scale variation detection model

Target objects in remote sensing images often exhibit significant scale variations. The potential interference between objects of different scales makes it challenging for models to accurately detect both small and large targets simultaneously.

Existing methods typically address this by extracting and integrating features from multiple levels, enabling models to effectively adapt to variations in target scales. Wang et al. [28] proposed Feature-Reflowing Pyramid Network(FRPNet), a feature-reflowing pyramid network that addresses large variations in object scales using a non-local block and pyramid structure. Huang et al. [29] proposed a Cross-Scale Feature Fusion Pyramid Network(CF2PN), which employs a Cross-Scale Fusion Module (CSFM) to enhance semantics and a Thinning U-shaped Module (TUM) to extract multi-level features, addressing the challenge of object scale variations in RSOD. These methods primarily extract and integrate multi-level features through CNN to improve model accuracy. However, CNN still faces challenges in extracting global information, which has led some studies to incorporate self-attention to overcome these limitations. Li et al. [30] proposed the Scale-Robust Complementary Learning Network (SCLNet), a network that enhances robustness against scale variations in RSOD through self-attention and interscale contrastive complementary learning. However, separating the self-attention from CNN for feature extraction often leads to insufficient feature fusion. To address this, researchers have further explored the integration of CNN and self-attention. Zhan et al. [21] combined CNN with Transformers, enhancing target localization performance through multi-scale visual features and multi-granularity textual embeddings.

Although existing methods have made some progress in addressing scale variations in RSOD by extracting and integrating multi-level features, they typically use CNN-based strided convolutions for downsampling, leading to the loss of object information. Moreover, these methods overlook the importance of explicit spatial priors in the self-attention mechanism and lack effective mechanisms to integrate features extracted by CNN and self-attention, which limits the model’s context-aware modeling capability. Recent wavelet-based studies provide new insights. The Wavelet-based Bi-dimensional Aggregation Network WBANet [31] employs Haar wavelet decomposition to achieve lossless downsampling preserving high-frequency textures while expanding receptive fields. The Dual Encoder Crack Segmentation Network DECS-Net [32] utilizes Haar wavelet-based high-low frequency attention to enhance edge awareness in structural damage detection. Despite these advances current methods still lack mechanisms to prevent information loss during downsampling incorporate explicit spatial priors in self-attention and fully unify CNN-local and Transformer-global features [33]. Complementing these efforts, evolutionary multi-objective optimization (EMO) studies reveal that explicit spatial priors critically stabilize model convergence under complex variations [34–37]. Williams et al. [34,37] independently demonstrated, through bi-objective optimization, how sparse adversarial attacks and verification of spatial constraints mediate the transformation of feasible regions—insights that align closely with our emphasis on spatially aware modeling. To address these issues, this paper designs three modules: LEM, HFDSA, and SIIM.

## Proposed method

This paper adopts YOLOv8 as the baseline model. Compared to the latest algorithms, YOLOv8 demonstrates stronger generalization capabilities on small-scale datasets and achieves higher accuracy on most datasets.

### Overview

**Fig 1:**
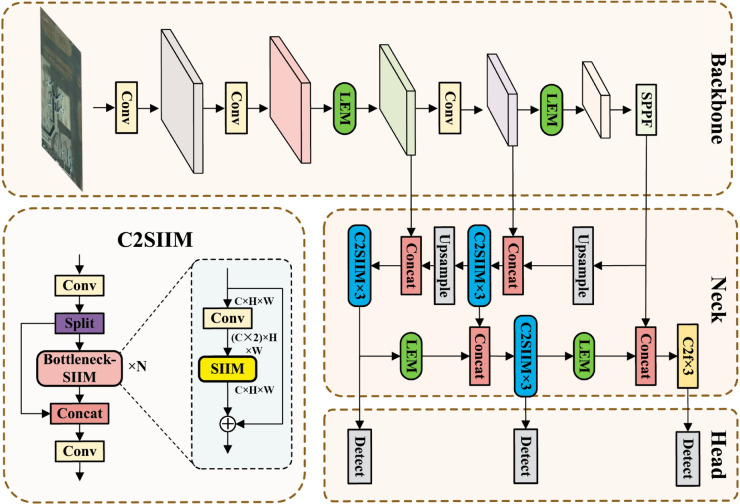
The overall framework of HWANet. The LEM replaces certain strided convolution modules in the backbone and neck networks to mitigate information loss during downsampling. Subsequently, the SIIM is utilized to construct C2SIIM, thereby enhancing the model’s context-aware modeling capability.

In summary, existing approaches suffer from two primary issues. First, strided convolutions lead to information loss during downsampling. Second, inadequate context-aware modeling impedes these methods from fully harnessing object information and associated features. These deficiencies collectively diminish detection performance in scenarios with substantial scale variations. Based on these considerations, this paper designs HWANet, with the overall architecture shown in Fig 1.

This paper proposes LEM to replace strided convolutions optimizing the model’s information retention during the downsampling process. To enhance the model’s context-aware modeling capability, this paper first proposes a low computational complexity HFDSA, which extracts global information by incorporating explicit spatial priors, thereby improving the model’s understanding of spatial structures in the image. Then, the proposed SIIM and HFDSA are integrated into C2SIIM to strengthen the interaction between features at different levels, promoting the deep fusion of global and local feature information. HFDSA and SIIM improve the model’s capability to leverage information from objects at various scales and their associated features, thereby enhancing the model’s ability to capture context-aware representations.

### Low-Frequency Enhanced Downsampling Module (LEM)

Most models rely on strided convolutions for downsampling. Strided convolutions reduce the dimensions of feature maps by skipping certain pixels, which can result in the loss of information. When dealing with large variations in object scales in RSOD, the use of strided convolutions can cause small objects to become blurred, and edge details of large objects may be lost.

**Fig 2:**
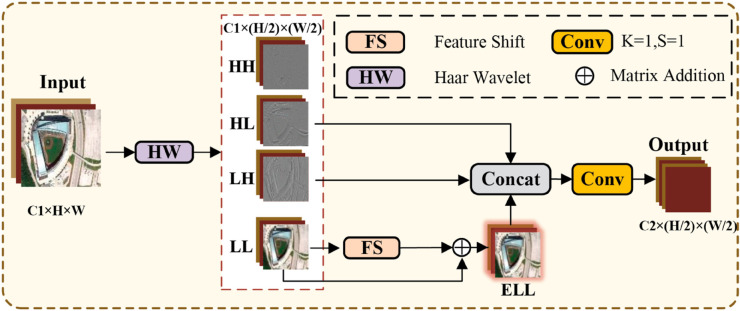
Structure of the LEM. LEM first performs a Haar wavelet transform on the input feature, discards the HH component, and simultaneously applies Feature Shift to the LL component to enhance feature representation, resulting in ELL. Finally, ELL, LH, and HL are concatenated along the channel dimension, and a 1 × 1 convolution is applied to perform channel transformation, producing the final output.

**Fig 3:**
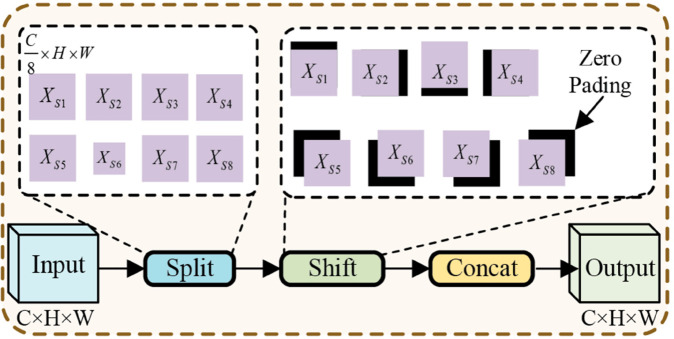
Structure of Feature Shift (FS). The Feature Shift mechanism divides the feature map into eight parts along the channel dimension and shifts each part by one position in a different direction. The missing areas are padded with zeros, and finally, all the shifted features are concatenated back together along the channel dimension.

To resolve this problem, this paper proposes LEM, whose specific structure is depicted in Fig 2. LEM mainly consists of the Haar wavelet transform and Feature Shift(whose structure is shown in Fig 3). These low-frequency components in the Haar wavelet transform reflect the overall shape and key features of the objects, while the high-frequency components typically contain noise. From this perspective, LEM first reduces the size of the feature map by applying the Haar wavelet transform to decompose the input feature map. Next, Feature Shift is applied to enhance the low-frequency components. Next, the high-frequency components are eliminated to minimize noise interference. Finally, the remaining frequency-domain feature components are concatenated along the channel dimension, and a 1×1 convolution is applied to reduce the number of feature channels, achieving downsampling and preventing information loss.

Given $Input\in {\mathbb{R}}^{C\times H\times W}$, the mathematical formulations of the LEM can be articulated as follows:

$$LL,LH,HL,HH=f_{HW}(\text{Input})$$ (1)

$$ELL=f_{FS}(LL)+LL$$ (2)

$$\text{Output}={\text{Conv}}_{1\times 1}(\text{Concat}(HL,LH,ELL))$$ (3)

where $LL,LH,HL,HH\in {\mathbb{R}}^{C\times \frac{H}{2}\times \frac{W}{2}}$ denote the approximation (low-frequency) component, vertical component, horizontal component, and diagonal component information obtained from the Haar wavelet transform, respectively. $f_{\text{HW}}$ and $f_{\text{FS}}$ denote the Haar wavelet transform and the Feature Shift operation (whose structure is shown in Fig 3), respectively. ELL represents the enhanced low-frequency components. In the LEM, this paper enhances the low-frequency components through the Feature Shift operation. Since remote sensing images typically contain a large amount of surface information, shifting features in different directions can capture diverse spatial features, providing a more comprehensive understanding of terrain, vegetation, buildings, and other land features, while also enhancing the model’s ability to capture fine spatial details. Finally, discarding the *HH* part can reduce noise interference, improve the purity of the feature, and highlight the key low-frequency component.

### Haar Frequency Domain Self-Attention (HFDSA)

Current approaches that utilize the self-attention mechanism often fail to account for the significance of explicit spatial priors when addressing scale variations in RSOD. While the self-attention mechanism is effective in capturing global information, the lack of explicit spatial priors makes it challenging for the model to accurately capture spatial relationships between objects of varying scales in remote sensing images, thus limiting its contextual modeling ability. Additionally, these methods have not effectively addressed the high computational complexity of the self-attention mechanism, which impacts the model’s efficiency and real-time performance.

**Fig 4:**
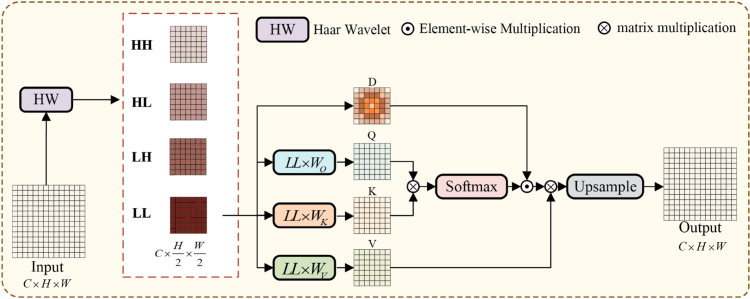
Structure of HFDSA. HFDSA first performs a Haar wavelet transform on the input features, then exclusively applies the self-attention to the LL component, and finally upsamples the processed features back to their original input size. During this process, a distance decay matrix is introduced to provide explicit spatial priors for the model. By processing only the low-frequency components, HFDSA effectively reduces noise interference and enhances the model’s stability.

Building on the previous points, this paper proposes a self-attention mechanism called the Haar Frequency Domain Self-attention (HFDSA), whose structure is shown in Fig 4. The HFDSA first applies the Haar wavelet transform to the input features to extract low-frequency components. These low-frequency components not only retain the main feature information of the image but also have a reduced size, significantly lowering the computational complexity.

Then, HFDSA employs the self-attention mechanism to process the low-frequency components, thereby extracting global information. During this process, a distance decay matrix is introduced to provide explicit spatial priors, thereby improving the model’s understanding of spatial structures within the image. Finally, the processed feature map is upsampled back to the original input size. The mathematical formulations of the HFDSA can be articulated as follows:

$$LL,LH,HL,HH=f_{HW}(\text{Input})$$ (4)

$$Q=LL\times W_{Q},\;K=LL\times W_{K},\;V=LL\times W_{V}$$ (5)

$$HFDSA=\left(\text{Softmax}(QK^{T})⊙D\right)V$$ (6)

where *LL*, *LH*, *HL*, *HH* represent the approximation (low-frequency) component, vertical component, horizontal component, and diagonal component information obtained from the Haar wavelet transform, respectively. $f_{\text{HW}}$ represents the Haar wavelet transform; *D* represents the distance decay matrix.

The HFDSA introduces a distance decay matrix *D*, which adjusts the attention weights based on the spatial distances between patches. Specifically, this matrix incorporates explicit spatial priors into the feature map, causing the attention weights between patches to decrease as their spatial distance increases. Explicit spatial priors effectively enhance the model’s understanding of spatial structures, thereby improving its ability to capture spatial relationships between objects, and in turn, enhancing its context-aware modeling capability.

The mathematical formulations of *D* can be articulated as follows:

$$\lambda =\ln\left(1-2^{-(j+c)}\right)$$ (7)

$$D_{x}=(\begin{array}{ccc} \left|x_{1}-x_{1}\right| & \cdots & \left|x_{n}-x_{1}\right|\\ \vdots & \ddots & \vdots \\ \left|x_{1}-x_{n}\right| & \cdots & \left|x_{n}-x_{n}\right| \end{array})$$ (8)

$$D_{y}=(\begin{array}{ccc} \left|y_{1}-y_{1}\right| & \cdots & \left|y_{n}-y_{1}\right|\\ \vdots & \ddots & \vdots \\ \left|y_{1}-y_{n}\right| & \cdots & \left|y_{n}-y_{n}\right| \end{array})$$ (9)

$$D^{\prime }=\lambda \times (D_{x}+D_{y})$$ (10)

$$D=(\begin{array}{cccc} D_{11} & D_{12} & \cdots & D_{1n}\\ D_{21} & D_{22} & \cdots & D_{2n}\\ \vdots & \vdots & \ddots & \vdots \\ D_{n1} & D_{n2} & \cdots & D_{nn} \end{array}),\;D_{ij}=\frac{e^{D_{ij}^{\prime }}}{\sum_{k=1}^{n}e^{D_{kj}^{\prime }}}$$ (11)

In remote sensing images, each patch has a unique coordinate representation (x,y) where *D* is the distance decay matrix generated on the basis of the coordinates of each patch. The decay factor *λ* is derived from the manually set initial value *j*, the number of input channels *c* in the HFDSA.

By integrating these improvements, HFDSA not only retains the advantages of the self-attention mechanism in extracting global information but also overcomes the computational bottlenecks and spatial relationship modeling deficiencies when handling large variations in object scales.

To alleviate the computational burden of self-attention, this paper applies the Haar wavelet transform to compress the input feature map, thereby reducing the overall computational complexity. Specifically, for an input sequence of length *N*, the self-attention mechanism typically has a computational complexity of $\text{O}({\text{N}}^{2})$. Let the Input be $Input\in {\mathbb{R}}^{C\times H\times W}$, and upon applying the Haar wavelet transform, $LL\in {\mathbb{R}}^{C\times \frac{H}{2}\times \frac{W}{2}}$ is obtained, where the computational complexity for both *Input* and *LL* is given by the following expressions:

$$n_{1}=O\left((H\times W{)}^{2}\right)`$$ (12)

$$n_{2}=O\left({\left(\frac{H}{2}\times \frac{W}{2}\right)}^{2}\right)=\frac{1}{16}O\left((H\times W{)}^{2}\right)$$ (13)

where *n*_1_ and *n*_2_ represent the computational complexity of the self-attention mechanism applied to the *Input* and *LL*, respectively. The results show that *n*_2_ is much smaller than *n*_1_.

### Spatial Information Interaction Module (SIIM)

**Fig 5:**
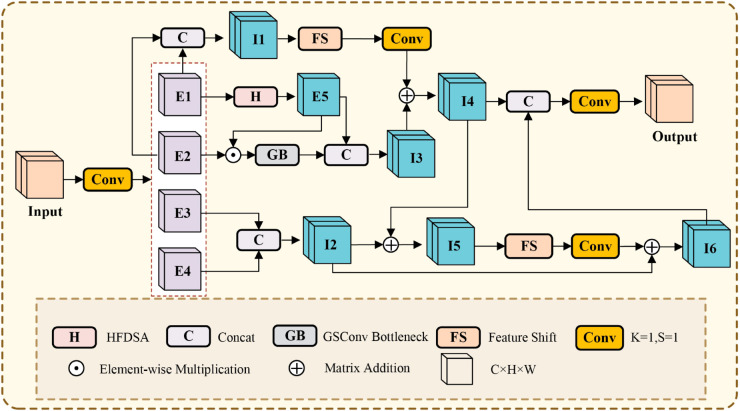
Structure of SIIM. SIIM first expands the input features along the channel dimension. Then, it processes E1 and E2 to obtain global information I4. Next, it extracts local edge information from E3 and E4 to obtain I6, with the guidance of I4 during this process. Finally, I4 and I6 are concatenated along the channel dimension, and the channels are compressed back to their original dimensionality.

In RSOD, because target objects often exhibit large variations in scale, it is particularly important to effectively interact and integrate information across different levels. Global information is used to capture overall features, while local information focuses on details. However, current models often overlook the connections and interactions between different levels. This lack of effective interaction can lead to feature conflicts or information loss, thereby weakening the context-aware modeling capability and preventing the models from fully utilizing the information from objects at different scales and their related features. To tackle these problems, this paper presents the spatial information interaction module (SIIM), the structure of which is shown in Fig 5.

**Fig 6:**
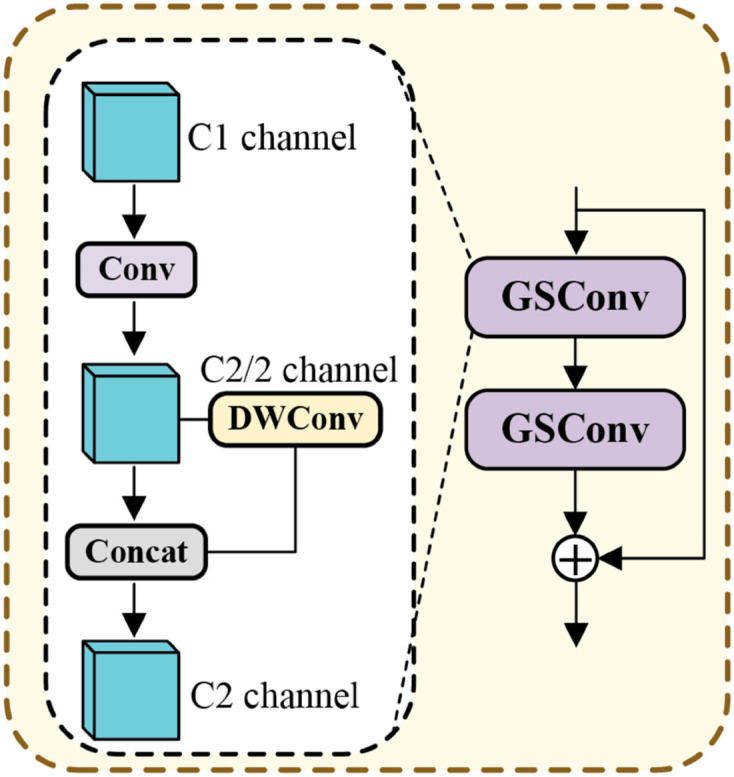
Structure of the GB. GSConv first applies a standard convolution to the input feature with C1 channels, reducing the channel number to C2/2, followed by a depthwise convolution (DWConv). Then, the output of DWConv is fused with the feature before processing, restoring the channel number to C2.

SIIM first performs a convolution operation to expand the channels of the input feature map, laying the foundation for subsequent processing, and then divides the feature map into *E*_1_, *E*_2_, *E*_3_, and *E*_4_. In the SIIM, HFDSA extracts global information from *E*_1_, resulting in the feature map *E*_5_. The computational complexity is significantly reduced by applying the self-attention mechanism to only a subset of channels. The global features captured by the self-attention mechanism, *E*_5_, are then multiplied element-wise with the feature map *E*_2_, which contains local details, effectively fusing the global and local information to form a more comprehensive representation with context awareness and detail resolution. After this multiplication, a lightweight GB (whose structure is shown in Fig 6) is used for information extraction. Then, the features processed by the GB are concatenated with *E*_5_, resulting in *I*_3_. The mathematical formulations can be articulated as follows:

$$E_{5}=HFDSA(E_{1})$$ (14)

$$I_{3}=\text{Concat}(GB(E_{2}⊙E_{5}),E_{5})$$ (15)

In SIIM, FS is applied to *I*_1_. Combining Feature Shift with 1 × 1 convolution, it effectively enhances the capture and fusion of the local features, making the model’s extraction of local information richer and more precise. Finally, the FS-processed feature maps are added to *I*_3_, resulting in the feature *I*_4_. The mathematical formulations can be articulated as follows:

$$I_{1}=\text{Concat}(E_{1},E_{2})$$ (16)

$$I_{4}={\text{Conv}}_{1\times 1}(f_{FS}(I_{1}))+I_{3}$$ (17)

FS is applied to *E*_3_ and *E*_4_ to extract local texture information, resulting in the feature map *I*_6_. The mathematical formulations can be articulated as follows:

$$I_{2}=\text{Concat}(E_{3},E_{4})$$ (18)

$$I_{5}=I_{2}+I_{4}$$ (19)

$$I_{6}=I_{2}+{\text{Conv}}_{1\times 1}(f_{FS}(I_{5}))$$ (20)

In extracting local texture features *I*_6_, this paper uses *I*_4_ for guidance, providing contextual support for the edge features, and preventing the edge and texture features from acting independently without a global semantic background.

SIIM performs channel concatenation and convolution on *I*_4_ and *I*_6_, resulting in the output feature map. SIIM effectively integrates multi-level information by interacting with information at different levels and exploring the relationships between them. This significantly improves the model’s context-aware modeling capability, allowing it to fully leverage the target objects and their associated information, thereby better handling large variations in object scales.

## Experimental evaluation and analysis

In this paper, YOLOv8n is chosen as the baseline model, and the performance of HWANet is assessed using the NWPU-VHR-10 and SAR-Airport-1.0 datasets, with an 80-20 split for training and testing. All experiments are carried out on an RTX 4060 GPU, utilizing the PyTorch framework. During the training, the input images are resized to 640x640 pixels. The model is trained for 300 epochs with a batch size of 4, using the SGD optimizer. The learning rate starts at 0.01, the weight decay coefficient is 0.005, and the momentum is initialized at 0.937.

### Datasets

This paper trains and tests HWANet on two public datasets. The following is a detailed introduction to these datasets:

- The NWPU VHR-10 [38–40] dataset is an open-access remote sensing dataset created for the purpose of spatial object detection. It consists of 800 ultrahigh-resolution images with object scales ranging from 0.1 to 30 meters, making it suitable for multi-scale detection. The backgrounds cover urban, rural, and industrial areas with complex scenes and varying lighting conditions, offering both challenges and practical value for training and evaluating object detection algorithms. Among these images, 650 are meticulously annotated, and the dataset includes diverse object categories such as airplanes (A), ships (S), storage tanks (ST), baseball fields (BF), tennis courts (TC), basketball courts (BC), ground track field (GTR), harbors (H), bridges (B), and vehicles (V). The dataset can be obtained at https://gcheng-nwpu.github.io/.
- The SAR-Airport-1.0 [41] dataset is a specialized, high-quality dataset created for object detection tasks in synthetic aperture radar (SAR) imagery. It contains SAR images from multiple airports, covering various environments and conditions, and provides rich object information such as airplanes, aprons, and runways. Each image’s objects are meticulously annotated, including bounding boxes and class information. The dataset can be obtained at https://doi.org/10.57760/sciencedb.15367.

### Accuracy metrics

In remote sensing object detection tasks, mAP50 and mAP50-95 are key metrics for evaluating model performance. mAP50 calculates the mean average precision at an intersection over a union (IoU) threshold of 0.5, which is suitable for scenarios with lower overlap requirements, such as detecting buildings in urban environments. In contrast, mAP50-95 encompasses multiple IoU thresholds ranging from 0.5 to 0.95, providing the average precision of the model under various overlap conditions. This multilevel evaluation offers a more comprehensive assessment of the model’s detection capability in complex backgrounds. By combining these two metrics, researchers can better understand the model’s performance and reliability in practical applications. The mathematical formulations of the mAP can be articulated as follows:

$$P=\frac{TP}{TP+FP}$$ (21)

$$R=\frac{TP}{TP+FN}$$ (22)

$$AP=\sum_{i=0}^{n-1}\left[R(i)-R(i+1)\right]\cdot P(i)$$ (23)

$$mAP=\frac{1}{n}\sum_{i=1}^{n}AP_{i}$$ (24)

In the formula provided, TP stands for the count of true positive samples that the model predicts correctly. FN indicates the actual positives that are mistakenly predicted as negatives (false negatives), while FP refers to the negative samples that the model inaccurately classifies as positives. P represents the model’s precision, R indicates recall, and n denotes the total number of classes involved.

### Comparisons with previous methods

This paper compares multiple models on the NWPU-VHR-10 dataset in terms of parameter count, GFLOPs, and object detection performance (mAP50 and mAP50-95). As shown in Table 1, the HWANet model has only 2.75M parameters—the fewest among all the compared models—and with the lowest computational complexity at just 7.90 GFLOPs. First, HWANet employs a downsampling module called the LEM, which retains important image features. Second, HWANet integrates SIIM and HFDSA, significantly enhancing the model’s context-aware modeling capability, which in turn improves its accuracy in detecting objects of various sizes. In terms of the mAP50-95 metric, HWANet achieves an accuracy of 61.0%, surpassing models with higher computational demands, such as Faster R-CNN. Although Faster R-CNN has a larger parameter count, its performance is limited by structural complexity and incompatibility with high-resolution remote sensing imagery. In contrast, WTHA-ViT [42] employs a wavelet tree structure to decompose features into frequency subbands, enabling adaptive frequency selection for each patch and facilitating cross-frequency interactions. By leveraging wavelet-based downsampling, it reduces computational load; however, its performance trails HWANet due to less efficient global context integration and higher parameter redundancy. Similarly, DetailCaptureYOLO [43] utilizes discrete wavelet transforms (DWT) for detail-preserving downsampling and a Dynamic Fusion PAN to aggregate multi-scale features, achieving superior mAP50-95 that reflects exceptional small-object detection capabilities—though at a 3.5× higher computational cost than HWANet. Collectively, this highlights that while TPH-YOLO’s advantage in mAP50-95 is built upon its higher parameter count and GFLOPs, HWANet establishes a unique balance of efficiency and accuracy for resource-constrained applications.

**Table 1:** Comparison Experiments for HWANet in NWPU VHR-10 Dataset.

Method	Para(M)	GFLOPs	mAP50	mAP50-95
Faster R-CNN [44]	41.17	127.7	77.8	–
RetianNet [45]	36.29	123.27	89.4	–
ShuffleNet [46]	12.1	82.17	83.0	–
ARSD [47]	11.57	26.65	90.92	–
Swin-Transformer [48]	29.98	79.1	86.8	52.5
YOLOv6S [49]	16.29	44.0	91.8	59.6
YOLOv8S	11.12	28.5	92.3	59.8
YOLO-World [50]	3.53	10.4	92.6	59.7
YOLOv10S [51]	8.04	24.5	90.4	57.9
TPH-YOLO [52]	41.53	160.1	92.9	57.6
FFCA-YOLO [26]	7.14	51.4	92.8	57.8
WTHA-ViT [42]	35.07	228.46	89.13	56.52
DetailCaptureYOLO [43]	28.7	109.3	84.14	54.61
HWANet (Ours)	**2.75**	**7.9**	**93.1**	**61.0**

**Fig 7:**
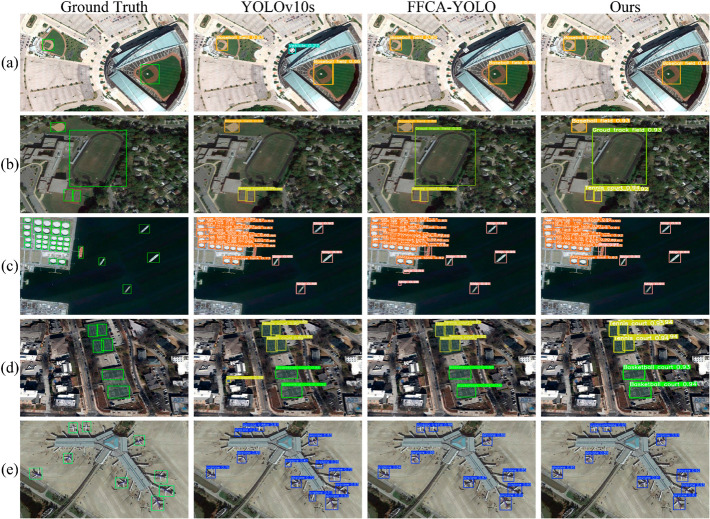
Detection results of each model. The partial detection results of YOLOv10S, FFCA-YOLO, and HWANet on the NWPU VHR-10 dataset are as follows: in (a) and (e), YOLOv10S incorrectly identified the background as a vehicle and an airplane, respectively; in (b), Yolov10S failed to accurately detect the ground track field; in (c), FFCA-YOLO mistakenly identified the background as a ship; and in (d), only HWANet correctly detected all the objects. Reprinted from [38–40] under a CC BY license, with permission from Gong Cheng, original copyright 2014. Data public visit: https://gcheng-nwpu.github.io/.

Fig 7 shows a comparison of the detection results of the YOLOv10s, FFCA-YOLO, and HWANet models on several images. The figure illustrates significant differences in the performance of the three models across different scenarios. In (a) and (b) of Fig 7, YOLOv10s results in false-positives and missed detections, whereas HWANet performs more accurately in bounding large objects such as baseball fields and ground track fields. In (d) of Fig 7, HWANet accurately detects objects even in complex backgrounds, demonstrating the advantages of its feature fusion strategy in RSOD. In general, HWANet demonstrates superior performance compared to the other models in terms of detection accuracy and bounding box precision.

**Fig 8:**
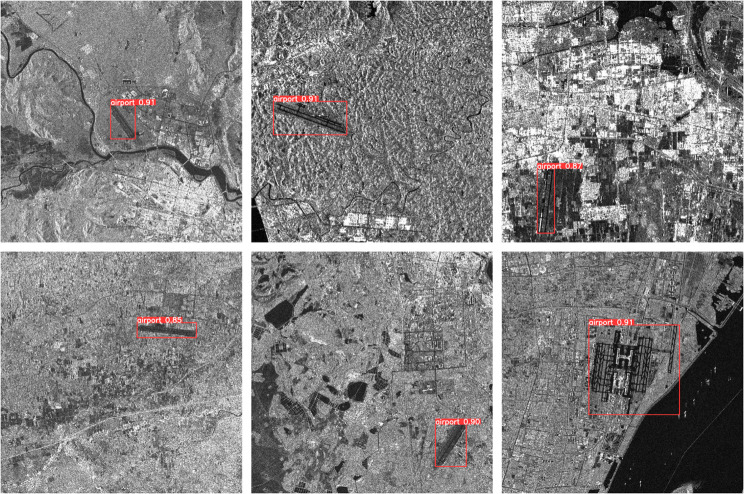
The detection results of SAR-Airport-1.0. Reprinted from [41] under a CC BY license, with permission from Fan Zhang, original copyright 2024. Data public visit: https://doi.org/10.57760/sciencedb.15367.

Table 2 presents a comparative analysis of multiple object detection models on the SAR-Airport-1.0 dataset, with Fig 8 illustrating some detection results from HWANet. The HWANet model, with only 2.75M parameters, has the smallest parameter count among all the models. Despite the lightweight design, HWANet achieves a mAP50 score of 99.1%, performing on par with larger models like TPH-YOLO (41.51M parameters). Expanding this comparison, WTHA-ViT leverages its wavelet tree structure to adaptively select frequency components for SAR imagery, achieving 96.6% mAP50 at 228.46 GFLOPs. Meanwhile, DetailCaptureYOLO’s discrete wavelet transform (DWT) downsampling and Dynamic Fusion PAN deliver the highest mAP50 (97.7%) among all models, demonstrating exceptional airport infrastructure detection capability. For comprehensive accuracy (mAP50-95), TPH-YOLO leads at 80.1% versus HWANet’s 77.3%, while WTHA-ViT and DetailCaptureYOLO attain 75.0% and 74.2% respectively. TPH-YOLO’s advantage stems primarily from its Transformer prediction head (TPH) handling complex scenarios. Notably, both wavelet-based models show tradeoffs: WTHA-ViT’s frequency-adaptive approach improves airport structure recognition but incurs higher computation, while DetailCaptureYOLO’s detail-preserving design excels in mAP50 yet trails in holistic mAP50-95 due to SAR-specific texture challenges.

**Table 2:** Comparison Experiments for HWANet in SAR-Airport-1.0.

Method	Para(M)	GFLOPs	mAP50	mAP50-95
Swin-Transformer [48]	29.98	79.1	98.1	72.5
YOLOv6S [49]	16.29	44.0	**99.1**	76.3
YOLOv8S	11.12	28.4	98.9	75.2
YOLO-World [50]	3.53	9.4	98.4	75.1
YOLOv10S [51]	8.03	24.4	95.8	69.7
TPH-YOLO [52]	41.51	160.6	**99.1**	**80.1**
FFCA-YOLO [26]	7.12	51.2	97.2	73.0
WTHA-ViT [42]	35.07	228.46	96.6	75.0
DetailCaptureYOLO [43]	28.7	109.3	97.7	74.2
HWANet (Ours)	**2.75**	**7.9**	**99.1**	77.3

### Ablation study of HWANet

To assess the performance of the proposed method, ablation studies were performed using the NWPU-VHR-10 dataset. The findings of these experiments are shown in Tables 3 to 7.

**Table 3:** Ablation Experiment on NWPU-VHR-10.

Method	Para(M)	GFLOPs	mAP50	A	S	ST	BF	TC	BC	GTR	H	B	V
Baseline	3.00	8.1	91.6	**99.5**	**94.7**	98.9	98.0	94.0	77.0	96.8	97.5	70.4	88.9
Baseline + LEM	**2.67**	**7.6**	92.4	98.3	92.4	98.9	**98.9**	**98.8**	**80.1**	**99.4**	95.6	74.4	86.9
Baseline + SIIM	3.08	8.4	92.7	99.4	92.8	97.4	**98.9**	96.3	75.3	99.3	**98.2**	82.7	87.0
Baseline + LEM + SIIM	2.75	7.9	**93.1**	99.4	92.3	**99.0**	**98.9**	95.4	78.2	96.2	97.3	**84.9**	**89.7**

**Table 4:** Ablation Experiment on NWPU-VHR-10 without HFDSA.

Method	Para(M)	GFLOPs	mAP50	A	S	ST	BF	TC	BC	GTR	H	B	V
Baseline +SIIM	3.07	8.4	92.0	99.4	**93.9**	87.4	98.3	93.4	**81.8**	97.0	96.2	**83.3**	89.2
Baseline +LEM + SIIM	**2.74**	**7.7**	**92.1**	99.4	92.1	**99.2**	**99.0**	**95.9**	76.7	**98.5**	**94.1**	75.0	**91.0**

**Table 5:** Ablation Experiment on NWPU-VHR-10 without HH.

Method	Para(M)	GFLOPs	mAP50	mAP50-95
HWANet+(HH)	2.81	8.0	90.6	57.9
HWANet (Ours)	**2.75**	**7.9**	**93.1**	**61.0**

**Table 6:** Ablation Experiment on NWPU-VHR-10 without the distance decay matrix.

Method	Para(M)	GFLOPs	mAP50	mAP50-95
HWANet-(D)	2.75	7.9	90.7	59.5
HWANet (Ours)	**2.75**	**7.9**	**93.1**	**61.0**

**Table 7:** Comparing the GPU Memory Usage of SIIM+HFDSA and SIIM+HFDSA (Without Using Haar Wavelet Transform).

Method	Input Size	GPU Memory Usage (MB)
SIIM+HFDSA(without HW)	64×32×32	65.19
SIIM+HFDSA	64×32×32	5.17
SIIM+HFDSA(without HW)	64×16×16	4.30
SIIM+HFDSA	64×16×16	0.54

Table 3 presents a comparison of the baseline model with several variants incorporating the LEM and SIIM modules on this dataset. The experimental results show that the inclusion of LEM and SIIM modules reduces both the number of parameters and the computational complexity of the model. Notably, the parameter count reaches its lowest value at 2.67M when only the LEM is introduced. The mAP50 of the baseline model is 91.6%, which increases to 92.4% with the introduction of the LEM, and further improves to 92.7% after adding SIIM. When both LEM and SIIM are applied simultaneously, the model achieves its highest mAP50 of 93.1%. The model’s performance in detecting various object categories also shows significant improvement. For example, in the ground track field category, the accuracy increases from 77.0% in the baseline model to 78.2% after incorporating LEM and SIIM, indicating notable enhancements in this category. The most substantial improvement occurs in the bridge category, where the baseline accuracy of 70.4% increases to 84.9% with the addition of LEM and SIIM, representing a 14.5% increase. This result demonstrates that the LEM module, by enhancing low-frequency feature extraction, enables the model to better capture objects such as bridges, which exhibit large spans and distinct structural characteristics. In addition, the SIIM module further enhances the model’s ability to identify bridge objects in complex scenes by integrating features from different levels. The combination of LEM and SIIM not only improves the overall detection accuracy but also ensures that the model maintains a low parameter count and computational complexity while delivering superior performance.

HFDSA demonstrates strong global information extraction capabilities. To more specifically highlight the importance of HFDSA, this paper provides a detailed analysis in Table 4, comparing the model’s performance on the NWPU-VHR-10 dataset after replacing HFDSA in the SIIM module with standard convolution. According to the data from Tables 3 and 4, when only SIIM is introduced, the model’s mAP50 drops from 92.7% (with HFDSA) to 92.0% (with standard convolution). Further analysis of the results with both LEM and SIIM shows that the mAP50 decreases from 93.1% to 92.1%. The results highlight the essential role of HFDSA in handling significant variations in object scales. By leveraging its global information extraction capability and incorporating explicit spatial priors, HFDSA enables the model to better understand spatial structures in images, thereby significantly improving detection accuracy.

The ablation study in Table 5 reveals that omitting the HH component from the LEM module is essential. When the HH branch is retained (HWANet+(HH)), mAP50 falls from 93.1% to 90.6% and mAP50-95 from 61.0% to 57.9%, while Para(M) and GFLOPs edge up from 2.75 M to 2.81 M and from 7.9 to 8.0, respectively. The performance drop confirms that the high-frequency diagonal subband mainly injects sensor artifacts and environmental noise, which corrupt learned features and undermine detection robustness. Moreover, its removal trims the representation without discarding semantically useful cues, since the minor parameter and computation reductions show that HH adds little discriminative information yet consumes resources. SIIM’s feature-shift mechanism compensates for any texture loss, allowing HWANet to deliver an optimal accuracy-efficiency balance.

To validate the contributions of the distance decay matrix and Haar wavelet-based low-frequency attention, we conducted an ablation study comparing the full HWANet with a variant that removed the distance decay matrix (HWANet-(D)). As shown in Table 6, both models maintained identical parameters (2.76M vs. 2.75M) and computational costs (7.9 GFLOPs), ensuring that any performance differences were solely due to architectural changes. Removing the distance decay matrix led to significant performance degradation (-2.4% mAP50, -1.5% mAP50-95). This matrix provides explicit spatial priors in the High-Frequency Spatial Attention (HFDSA) mechanism, forcing attention weights to decay with increasing spatial distance. Without it, the model struggled to capture spatial relationships between multi-scale objects, weakening the context-aware modeling capability. Ultimately, the synergy between the distance decay matrix and wavelet-based attention enabled HWANet to achieve peak performance (93.1% mAP50). The wavelet attention provided an efficient and noise-robust foundation for global modeling, while the distance decay matrix injected critical spatial inductive biases for precise relationship reasoning across scales. This combination is essential for handling extreme scale variations in Remote Sensing Object Detection (RSOD).

**Fig 9:**
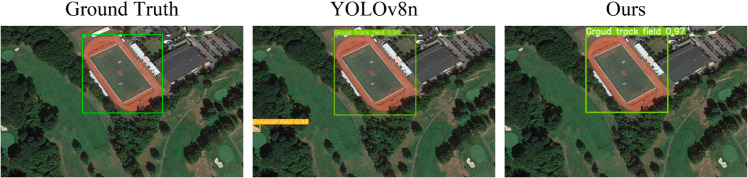
Comparison of the detection results between YOLOv8n and HWANet. Reprinted from [38–40] under a CC BY license, with permission from Gong Cheng, original copyright 2014. Data public visit: https://gcheng-nwpu.github.io/.

Fig 9 visualizes the comparison between HWANet and YOLOv8n on a specific image. YOLOv8n mistakenly detected the background as a baseball field. Owing to the powerful global information extraction capability of HFDSA, and the effective information interaction of SIIM, HWANet avoids this misdetection, delivering more accurate results.

Table 7 compares the GPU memory usage between SIIM+HFDSA and SIIM+HFDSA(without HW) on tensors of different sizes. The results show that, for the 64 × 32 × 32 input size, SIIM+HFDSA demonstrates a significant advantage, with a memory usage of only 5.17 MB, much lower compared to SIIM+HFDSA(without HW)’s 65.19 MB. However, for the 64 × 16 × 16 input size, the GPU memory usage of SIIM+HFDSA(without HW) increases to 4.3 MB, significantly higher than SIIM+HFDSA’s 0.54 MB. This is because the self-attention mechanism requires calculating the correlations between each patch, leading to increased computational and memory demands. In contrast, HFDSA compresses the matrix representation through the Haar wavelet transform, reducing memory consumption while retaining the main image information.

## Conclusion

This paper addresses the issues of information loss during downsampling and insufficient context-aware modeling capability in existing methods for RSOD when dealing with large variations in object scales. A Haar wavelet-based Attention Network, HWANet, is proposed. The model consists of three main modules: the Low-frequency Enhanced Downsampling Module (LEM), the Haar Frequency Domain Self-attention Mechanism (HFDSA), and the Spatial Information Interaction Module (SIIM). The LEM utilizes Haar wavelet transform to perform downsampling on feature maps and enhances the low-frequency components through Feature Shift (FS) operations. This approach effectively preserves critical information while preventing the loss of information from objects at different scales during the downsampling process. In addition, to further enhance the model’s context-aware modeling capabilities and make more effective use of target information, this paper introduces the HFDSA and SIIM modules. HFDSA incorporates explicit spatial priors to enhance the model’s understanding of spatial structures within images. SIIM facilitates the interaction and fusion of features at different levels, enabling the effective integration of multi-level features.

Despite these advances, HWANet presents certain limitations worthy of consideration. The method’s reliance on Haar wavelet transforms may impact its adaptability to complex scenes with non-stationary texture patterns. Additionally, the multi-module architecture could introduce computational demands challenging for edge device deployment. Future work will address these constraints through lightweight wavelet alternatives and dynamic feature pruning strategies.

In future research, we aim to combine HWANet with more sophisticated self-supervised or semi-supervised learning techniques to further improve its detection performance on small-sample or unlabeled datasets. Moreover, to address more challenging remote sensing scenarios, we will explore the incorporation of more sophisticated semantic fusion strategies into HWANet to further improve its performance in RSOD.
